# Supplementary material for: Giant strain control of magnetoelectric effect in Ta|Fe|MgO
Source: Sci Rep. 2016 Sep 6;6:32742. doi: 10.1038/srep32742 (PMC5011689; doi:10.1038/srep32742)
Supplement: Supplementary Information [file srep32742-s1.pdf]

Supplementary Information

Giant strain control of magnetoelectric effect in Ta/Fe/MgO

Dorj Odkhuu

Department of Physics, Incheon National University, Incheon 406-772, Republic of Korea

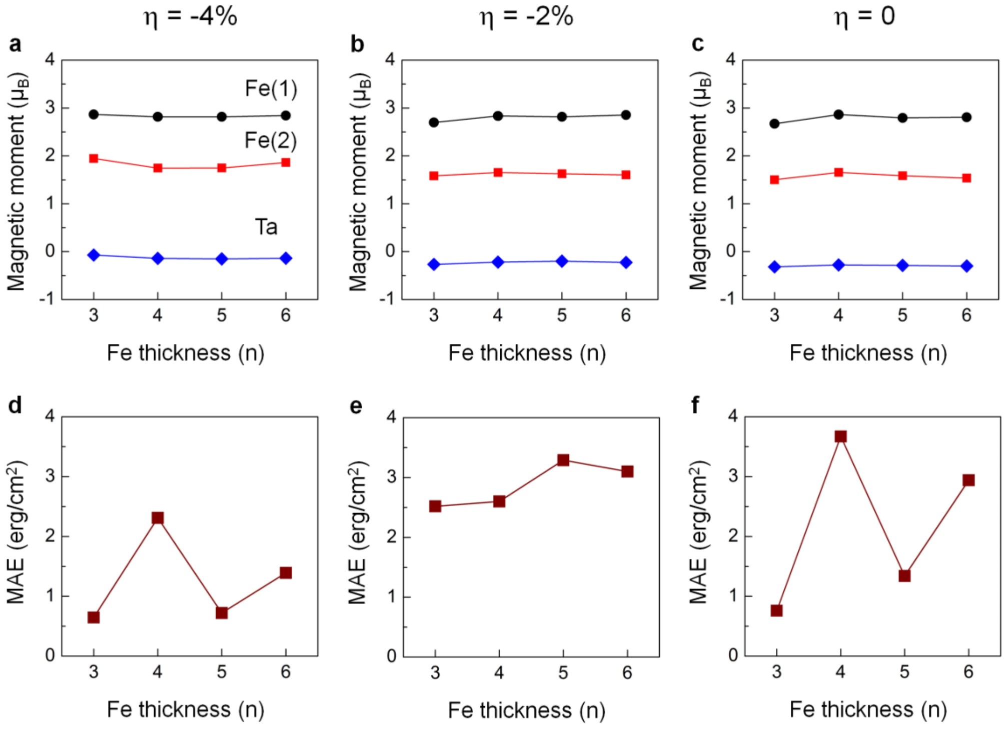

Figure S1. Fe-thickness-dependent magnetic moments of Fe(1) (circle) at the Fe/MgO, and Fe(2) (square) and Ta (diamond) atoms at the Ta/Fe interface of Ta/Fe/MgO for (a) η = –4%, (b) η = –2%, and (c) zero strain. Fe-thickness-dependent MAE of Ta/Fe/MgO for (d) η = –4%, (e) η = –2%, and (f) zero strain.

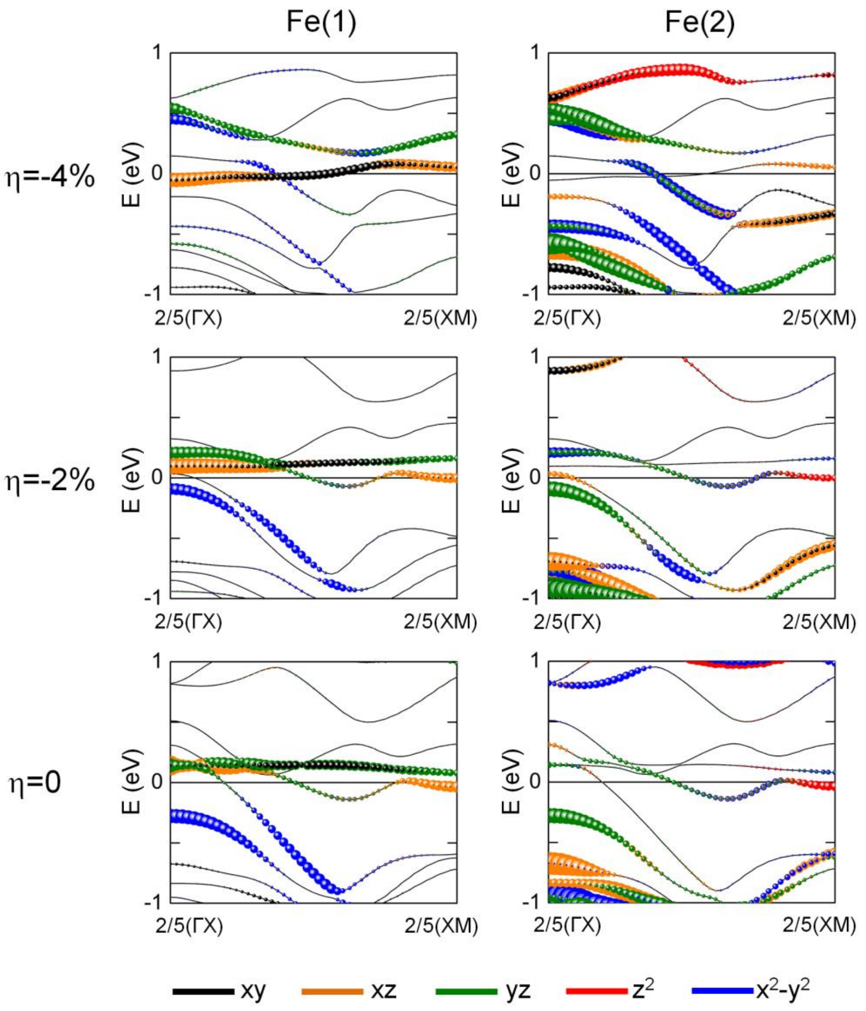

Figure S2. Minority-spin band structures along the 2/5(GX)–2/5(XM) line of Fe(1) at the Fe/MgO interface and Fe(2) at the Ta/Fe interface of Ta/Fe/MgO for η = –4%, η = –2%, and zero strain. The orbital states d_{xy}, d_{xz}, d_{yz}, d_{z^2}, and $d_{x^2-y^2}$ are denoted in black, orange, green, red, and blue, respectively. The symbol size represents the weight of the *d* orbitals. The Fermi level is set to zero energy.

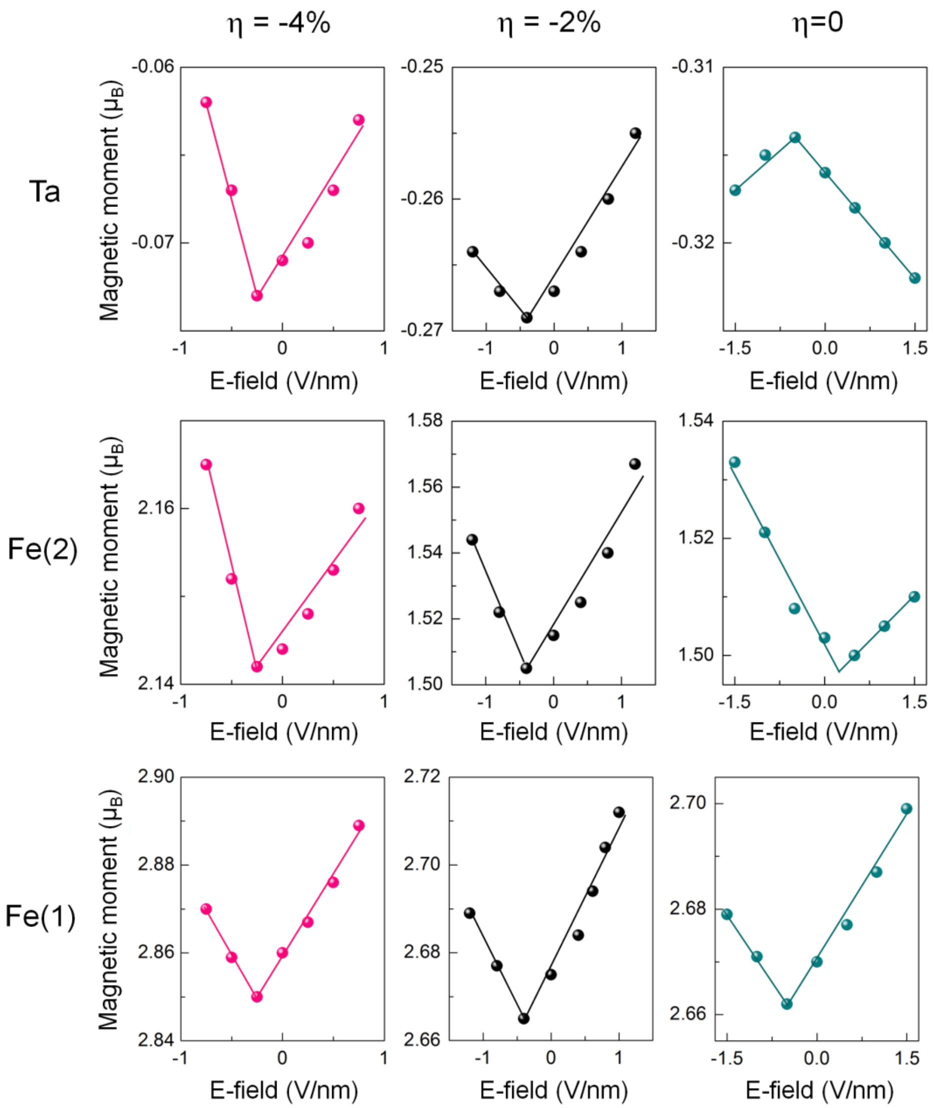

Figure S3. Electric-field dependence of Ta (topmost), Fe(2) (middle), and Fe(1) (bottommost) moments of Ta/Fe/MgO for η = –4% (left), η = –2% (center), and zero strain (right). The data points with circle symbol are fitted by a linear in all panels. The value of electric-field in the horizontal axis is that in MgO.

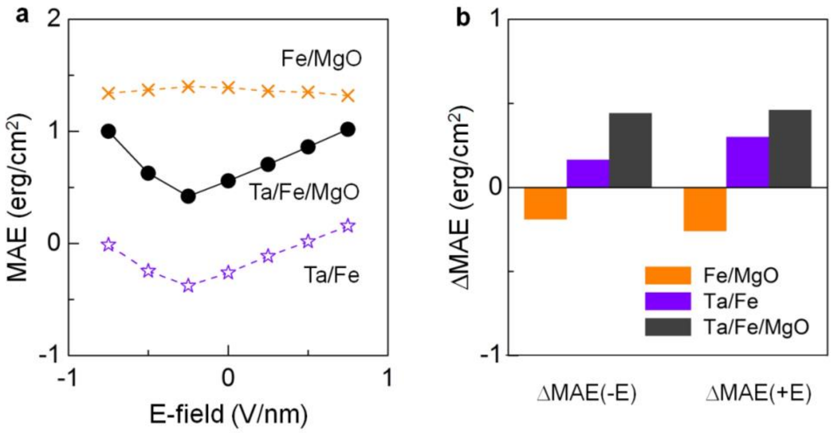

Figure S5. (a) Electric-field dependence of MAE for the individual Fe/MgO (orange cross) and Ta/Fe (violet star) interfaces. Results for Ta/Fe/MgO are also shown in black circles for comparison. (b) Electric-field-induced MAE, ΔMAE(±E) = MAE(±E) – MAE(0), for the individual Fe/MgO (orange) and Ta/Fe (violet area) interfaces. Results for Ta/Fe/MgO are also shown in dark-gray area for comparison.
